# Microneedle delivery systems for vaccines and immunotherapy

**DOI:** 10.1002/smo.20240067

**Published:** 2025-04-25

**Authors:** Haiyao Jia, Jinyuan Liu, Mengqian Shi, Manzar Abbas, Ruirui Xing, Xuehai Yan

**Affiliations:** ^1^ State Key Laboratory of Biopharmaceutical Preparation and Delivery Institute of Process Engineering Chinese Academy of Sciences Beijing China; ^2^ University of Chinese Academy of Sciences Beijing China; ^3^ Department of Chemistry Khalifa University of Science and Technology Abu Dhabi United Arab Emirates; ^4^ Center for Mesoscience Institute of Process Engineering Chinese Academy of Sciences Beijing China

**Keywords:** delivery systems, infectious disease prevention, microneedles, transdermal immunization, tumor therapy, vaccines

## Abstract

Microneedles (MNs) offer a precise and minimally invasive platform for delivering vaccines and therapeutic agents directly into the skin, leveraging the abundance of tissue‐resident immune cells to elicit robust and durable immune responses. Compared to traditional intramuscular or subcutaneous vaccination methods, MN‐based vaccines demonstrate superior patient compliance, enhanced antigen stability, and heightened immunogenicity, positioning them as a promising tool in biomedical applications. This review provides a comprehensive overview of the materials and fabrication techniques used in MN preparation, explores their structural classifications, and examines the role of antigens and adjuvants in optimizing vaccine efficacy. Furthermore, the diverse applications of MN delivery systems in preventing infectious diseases, advancing tumor immunotherapy, and addressing other immune‐related conditions are discussed.

## Graphical abstract

This paper reviews the preparation methods and materials of microneedles for drug delivery, summarizes the structures and types of microneedle systems, analyzes the influence of antigens and adjuvants on the efficacy of microneedle vaccines, and reviews the current application status of microneedles in the fields of vaccines and immunotherapy by listing microneedle vaccines used for the prevention or treatment of different diseases.

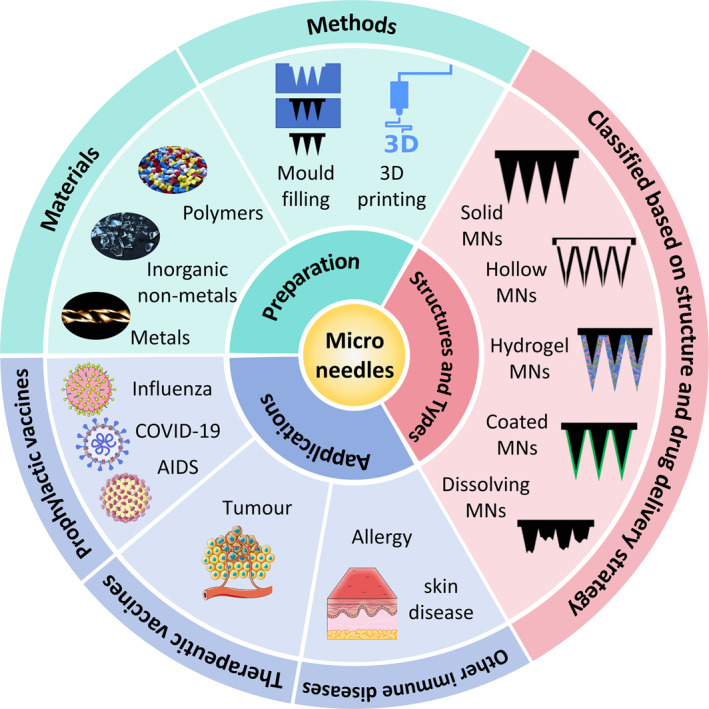

## 1 INTRODUCTION

Vaccines are biological agents designed to elicit specific immune responses through the introduction of antigens, playing a pivotal role in disease prevention and treatment. However, current popular intramuscular vaccines have poor compliance, as pain and fear associated with injections may lead approximately 10% of the population to avoid vaccination.^[1]^ Additionally, most traditional liquid formulations lack thermal stability and cannot be stored long‐term at room temperature (25°C), necessitating cold‐chain transportation.^[2]^ Furthermore, in developing countries requiring mass vaccination, limited vaccine production capacity often results in insufficient supply, making dose‐sparing strategies a critical consideration.^[3]^ Transdermal immunization, particularly via microneedle (MN) based vaccine delivery systems, offers a promising alternative by enabling a targeted, painless intradermal immune response with enhanced efficacy and patient acceptance.

**FIGURE 1:**
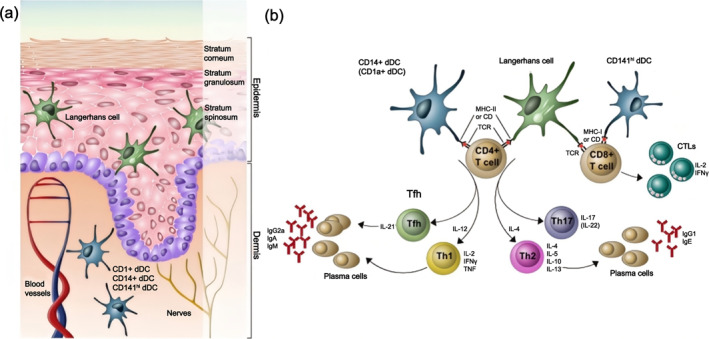
(a) Professional antigen‐presenting cells on the skin surface. (b) Processes of antigen presentation and triggered immunity in dermal dendritic cells and Langerhans cells.^[6]^ Copyright 2015, Elsevier Ltd.

MNs are a new delivery system of transdermal drug, which is composed of a substrate and an array of needles of a few hundred microns, typically ranging from 100 to 1000 μm.^[4]^ These structures enable precise delivery of drugs, vaccines, and other biologics directly into the skin tissue, bypassing the traditional barriers associated with oral or injectable administration. By facilitating drug transport through the skin's permeable layers and into the bloodstream, MNs can alter the drug's pharmacokinetic profile, enhancing both absorption efficiency and therapeutic efficacy.^[4]^ In addition, the skin is a highly immunocompetent organ containing many specialized antigen‐presenting cells (APCs), such as Langerhans cells (LCs) and dermal dendritic cells (dDCs).^[5]^ These APCs mature and migrate to lymph nodes, presenting and cross‐presenting exogenous antigens to CD4^+^ and CD8^+^ T cells, respectively, to activate subsequent immune responses (Figure 1).^[6]^ Therefore, MNs, a transdermal route of immunization, elicit a stronger immune response.

Due to the unique advantages of MNs, their application in the field of vaccines and immunization has received extensive attention. There is considerable evidence that MN vaccines are more immunogenic (the ability of substances such as pathogens, vaccines, or proteins to trigger responses from the immune system) than intramuscular vaccines. Notably, they require significantly lower doses to achieve comparable protective effects, which in turn minimizes vaccine‐associated toxicity.^[7]^ Moreover, MNs offer a significant advantage in enhancing vaccine stability, often eliminating the need for cryogenic storage and transportation. The COVID‐19 mRNA MN vaccines fabricated by Straeten et al. can be stably stored at room temperature for 6 months^[8]^ Besides, the MNs offer a minimally invasive and virtually painless vaccination method, enabling self‐administration or application with minimal training by simply pressing them onto the skin for a specified duration, thus reducing reliance on healthcare professionals.

This review provides a concise overview of the preparation methods and materials employed in MN systems for drug delivery. It summarizes the structural designs and classifications of MN platforms, analyzes the impact of antigens and adjuvants on the efficacy of MN‐based vaccines, and highlights the current advancements in MN applications within vaccination and immunotherapy. Furthermore, representative examples of MN vaccines developed for the prevention and treatment of various diseases are discussed to illustrate their therapeutic potential. Transcutaneous immunization using MN systems is expected to develop new therapeutic strategies for the prevention of infectious diseases or the treatment of tumors, with great potential for clinical translation.

## 2 PREPARATION OF MICRONEEDLES

MNs have emerged as versatile tools in drug delivery, medical aesthetics, and various biomedical applications. The design and fabrication of MNs are highly application‐specific, with material selection playing a critical role in ensuring functionality and biocompatibility. The primary materials used in MN fabrication include metals, inorganic non‐metals, and polymers, each chosen based on the intended application and performance requirements. Additionally, two predominant fabrication techniques—mold filling and additive manufacturing (3D printing)—are widely employed in current research. This review will provide a concise overview of these materials and fabrication methods, highlighting their relevance to specific biomedical applications.

### 2.1 Preparation materials

#### 2.1.1 Metals

MNs need to effectively penetrate the epidermis to deliver therapeutic agents into the skin (penetrate the 10–15 μm thick drug diffusion barrier, the stratum corneum, into the dermis and deliver the drug to the dermal microcirculation)^[9]^ without breaking or deforming during insertion. This requires a combination of high mechanical strength and optimal elasticity to withstand insertion forces while maintaining structural integrity. Given these mechanical demands, metallic materials emerge as a robust and reliable choice for MN fabrication.

Stainless steel and titanium alloys, commonly used as implant materials in the medical field, have excellent physical properties. SUS316L stainless steel, which is widely used in medical implantable devices, has a Young's modulus (a physical measure of a material's ability to resist deformation; the larger the value, the harder the material is to be stretched or compressed) of around 180 GPa,^[9]^ and the commonly used Ti‐6Al‐4V type titanium alloy has a yield strength of up to 795 MPa.^[10]^ It has been shown that the MNs prepared with stainless steel materials can still overcome the skin elasticity and penetrate the skin for many times at a speed of 3 m/s.^[11]^ The titanium MN patches still have 90% MNs penetration through the stratum corneum to complete drug delivery under the condition of drug coating.^[12]^ MNs prepared from stainless steel and titanium alloys usually have good mechanical properties.

In recent years, in addition to these metallic materials, which are already commonly used in the medical field, a number of new metallic materials with unique properties are being attempted for the preparation of MNs. Liquid metals have unique fluidity, excellent electrical conductivity and good biocompatibility, and MNs prepared with them have strong fixation capabilities and can also be used to stimulate wound healing by using the electrical signals generated by the conductivity of liquid metals.^[13]^ Silver nanoparticles have good antimicrobial properties. Preparation of self‐sterilizing MN patches with potent antimicrobial activity can be achieved by loading nano‐silver.^[14]^

#### 2.1.2 Inorganic non‐metals

Compared to metallic materials, inorganic non‐metallic materials offer greater ease of processing and shaping. Although their mechanical properties are generally inferior to those of metals, they still possess sufficient structural characteristics to enable effective skin penetration, making them promising candidates for biomedical applications. Therefore, inorganic non‐metallic materials are also often used in the preparation of MNs in order to simplify the process of MNs preparation for industrial mass production of MNs.

Silicon is a biocompatible and biologically inert material commonly used in MNs preparation. With the continuous development of the semiconductor field, the processing method of silicon has become simpler and more convenient, which also provides convenience for the application of silicon in the field of MNs. Solid and hollow silicon MNs can be prepared using a single step wet etch process and the MNs are capable of efficiently delivering drugs into the skin.^[15]^ In addition, the mechanical strength of silicon is sufficient for the successful insertion of the MNs into the skin. It has been shown that the axial force on silicon MNs when inserted into the skin is much less than its fracture limit.^[16]^

Ceramics and quartz glass can also be used to prepare MNs for drug delivery. Both ceramics and glass can be quickly produced in the desired shape, but both are brittle materials that may break during skin piercing. Some studies have shown that ceramic MNs prepared by micro‐nanofabrication techniques are partially broken during manual application.^[17]^ Similarly, while quartz glass MNs have many capabilities in laboratory research, they are subject to a variety of conditions when applied in the clinic.^[18]^ Therefore, ceramic and quartz glass MNs have slightly fewer applications in the medical field than silicon MNs.

#### 2.1.3 Polymers

Polymers represent a highly promising class of materials for the fabrication of MNs and have become a central focus of research in the field of MN materials science. Polymers have excellent biocompatibility and are biodegradable compared to the above two materials. Although their strength may be relatively low, their exceptional toughness, low cost, ease of operation, and other advantageous properties provide them with significant competitive potential in the field. Moreover, some of the polymers used for MNs preparation will themselves have certain biofunctional properties.

Naturally degradable macromolecules of biological origin are an excellent material for MNs. Hyaluronic acid is a natural component of the skin and is non‐immunogenic. After entering the skin in the form of MNs, it can be dissolved in the interstitial fluid to release its loaded drugs or used in medical cosmetology.^[19]^ Chitosan is also a non‐toxic natural polymer commonly used in the biomedical field, mostly in the preparation of hydrogels. The structure and type of MNs prepared with chitosan can be changed by adjusting the viscosity.^[20]^

Compared with natural polymers, the MNs prepared from synthetic polymers have higher strength and greater plasticity. It has been shown that the compressive strength of PVP MNs can reach 284 KPa with sufficient flexibility and strength.^[21]^ In addition, the researchers found that chitosan alone used in the preparation of MNs will make the drug release too fast and not easy to control, but the addition of PVA can make the MNs better form rigid structures, improve the mechanical strength and make the drug release more controllable.^[22]^ Therefore, the combination of materials may make MNs have better properties, which is also worth exploring.

### 2.2 Preparation methods

#### 2.2.1 Mold filling

Mold filling is a commonly used method of MNs preparation which is popular for MNs preparation as this method is easy to operate, reusable and can be applied to mass production. Mold filling needs to centrifuge or adjust the pressure to make the prepared material into the mold, and then remove the excess material through drying, irradiation, and other ways to shape the MNs.^[23]^ The MNs produced by mold filling have uniform dosage and regular shape, and can be used as a carrier for vaccine delivery.^[24]^

**FIGURE 2:**
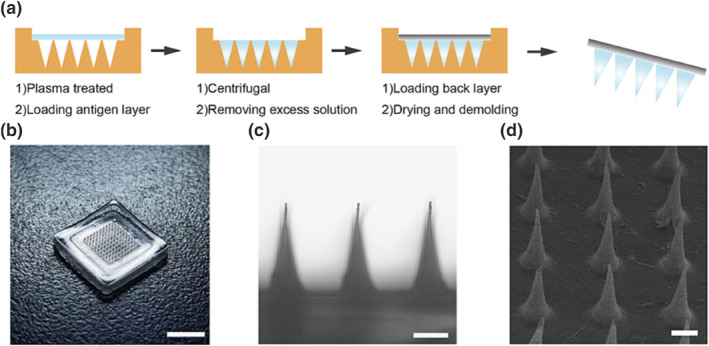
(a) Schematic illustration of the fabrication process of MNs. (b) Photograph of the MNs array (Scale bar, 1 cm). (c) Optical image of the magnified needles (Scale bar, 300 μm). (d) SEM image of MNs (Scale bar, 300 μm).^[25]^ Copyright 2023, Elsevier B.V.

With the deepening of research in the field of MNs, the method of mold filling is also constantly improved. To enhance the filling effect, the surface of the mold can be modified (Figure 2).^[25]^ The preparation of MNs can be carried out in a step‐by‐step manner, which allows the drug or vaccine to be pooled at the tip of the needles,^[26]^ and prevents the drug from being wasted, thus reducing the cost of the preparation. Although more and more methods of MNs preparation are being proposed and applied, the mold filling method still accounts for a large proportion of the MN delivery system field.^[23]^

#### 2.2.2 3D printing

3D printing is an emerging manufacturing technology that has been widely used in a variety of fields including the aerospace industry, production agriculture, and biomedicine.^[27]^ This technique offers the advantages of high resolution, high accuracy and customisability, which facilitate the preparation of MNs with different structures. Currently, the preparation of MNs that can deliver drugs, vaccines or live cells by 3D printing is also a research hotspot.

**FIGURE 3:**
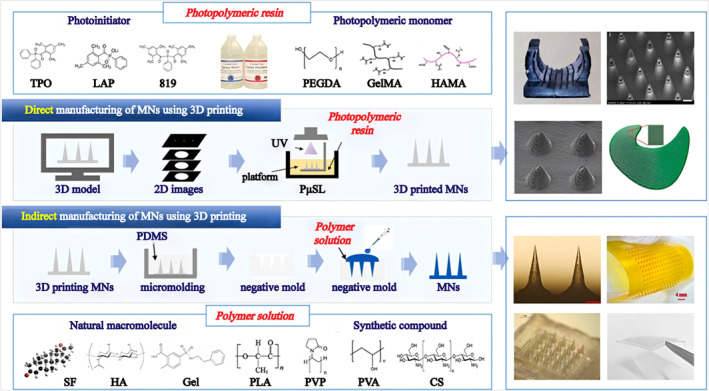
Flow chart of direct and indirect manufacturing of MNs using 3D printing.^[30]^ Copyright 2024, Elsevier B.V.

However, due to the small size of the MNs, this places high demands on the machines and inks used for 3D printing. Due to the low resolution of most current 3D printing machines, it is difficult to form a sharp tip when printing normally. To solve this problem, choo et al. adjusted the angle of printing and found that an optimal MN with a tip diameter of 30.2 ± 3.4 μm was obtained when the printing angle was 60°.^[28]^ For the same problem, Luzuriaga et al. used chemical etching to adjust the shape of the MNs after 3D printing.^[29]^ Recently, with the development of ultrafast pulsed laser technology, ultra‐high‐precision 3D printing technology has been developing rapidly, making it possible to precisely manufacture the tips of MNs (Figure 3).^[30]^ Therefore, 3D printing technology is expected to be widely used in the field of MNs manufacturing.

## 3 MICRONEEDLES STRUCTURES AND TYPES

**FIGURE 4:**
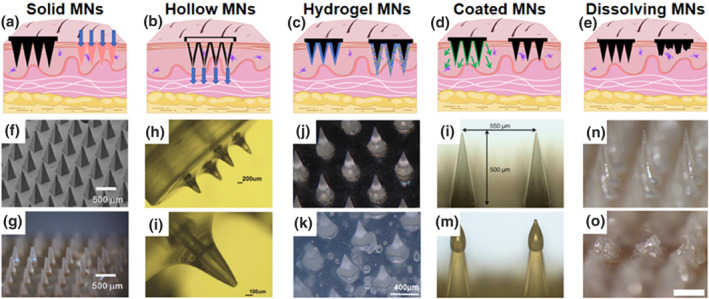
(a–e) Schematic diagrams of the five types of MN modes of action. (f, g) Solid MNs under scanning electron microscope (f) and optical microscope (g).^[31]^ Copyright 2010, Elsevier B.V. (h, i) Optical images of hollow MNs.^[32]^ Copyright 2022, Elsevier B.V. (j, k) Stereo‐micrographic images of hydrogel MNs before (j) and after (k) swelling.^[33]^ Copyright 2024, Elsevier B.V. (l, m) Images of the tip of MNs before (l) and after(m) coated.^[34]^ Copyright 2013, Elsevier Ltd. (n, o) Dissolving MNs before (n) and after (o) dissolution Optical microscopy images.^[35]^ Copyright 2021, Elsevier B.V.

The function of MNs is related to their structure and type, and MNs usually have different structures for different purposes (Figure 4). They can be classified into five categories based on their structure and drug delivery strategy: solid MNs, hollow MNs, hydrogel MNs, coated MNs and dissolving MNs, and they have their own advantages, disadvantages and applications (Table 1).

**TABLE 1:** Advantages, disadvantages and applications of five types of MNs.

MNs type	Advantages	Disadvantages	Applications
Solid MNs	High mechanical strength with strong skin penetration Separation of preparation and delivery minimizes impact on drug activity	Complex usage steps Slower drug release	Commonly used in the field of cosmetology
Hollow MNs	High drug‐loading capacity. Controlled drug delivery rate and dosage Can sample interstitial fluid via capillary action	Low mechanical strength Needle holes are easily blocked	Commonly used for delivery of large molecule drugs (e.g., insulin, vaccines)
Hydrogel MNs	Good biocompatibility. Suitable for slow drug release	Low mechanical strength Complex preparation process	Commonly used for long‐term drug release, (e.g., hormones, antibiotics, etc.)
Coated MNs	Simple preparation and low cost. Fast drug release	Limited drug‐loading capacity The preparation process may affect drug effectiveness	Suitable for topical treatments and rapid drug release
Dissolving MNs	MN dissolution after use, eliminating the need for removal and reducing infection risk Controlled and complete drug release	Low mechanical strength with limited penetration depth High preparation cost	Commonly used for vaccination and treatment of skin diseases

Solid MNs do not load with drugs or vaccines. They are pressed to puncture the MNs into the skin and form conduits to increase the skin permeability of drugs or vaccines (Figure 4a,f,g).^[31]^ Permeability is related to the insertion force, residence time and MNs density.^[36]^ After solid MNs pretreatment, biological agents such as drugs are applied to the skin where they will diffuse into the skin through the MN‐induced pores.^[37]^ Previous studies have shown that with the aid of solid MNs, drugs permeability of the skin is substantially increased and large model molecules, such as antibodies, can be successfully delivered.^[38]^ Since the preparation and drug delivery of solid MNs are done in steps, there is no need to consider inactivation of the drugs or vaccines during the manufacturing process. However, due to the complexity of its use steps, they are mostly used in the cosmetic field and less used for the delivery of drugs or vaccines.^[39]^

Hollow MNs have large‐capacity cavities that can be loaded with drugs or vaccines, so they can be used for the delivery of large doses of drugs or vaccines.^[40]^ In addition, hollow MNs can deliver drugs or vaccines in an automated manner, similar to a small syringe, which allows for precise delivery of medication (Figure 4b,h,i).^[32]^ They also allow for drugs flow rate control, and they have been shown that their delivery rate is correlated with parameters such as the geometry of the MNs and the viscosity of the drugs or vaccines.^[41]^ In addition to drug delivery, hollow MNs allow interstitial fluid sampling by capillary action.^[42]^ However, hollow MNs often suffer from limited mechanical strength and are prone to needle tip clogging, which can compromise their reliability and efficiency in practical applications.^[43]^

Hydrogel MNs are synthesized from a hydrogel polymeric matrix. The MNs are water‐absorbent and soluble, and when inserted into the skin, they dissolve by absorbing interstitial fluids which creates pores to release the drugs (Figure 4c,j,k).^[33]^ Due to the large pore size structures inside the hydrogel MNs, they can achieve high drug loading rates.^[44]^ In addition, these MNs are highly flexible in size and shape and can be removed from the skin intact.^[45]^ Similar to hollow MNs, hydrogel MNs can also be sampled from the body.^[46]^ Hydrogel MNs have porous structures that can be enriched for internal cell populations in skin tissue and brought out for in vitro monitoring.^[47]^

Coated MNs, in which drugs or vaccines are coated and immobilized on the surface of MNs, can be used to deliver a wide range of biologics with a simple fabrication process and fast delivery speed. Coated MNs immobilize the liquid formulation on the surface of the MNs into the solid formulation, which can increase the stability of the loaded vaccines.^[48]^ However, phase transitions can lead to varying degrees of reduction in vaccine efficacy. Therefore, it is crucial to employ preparation methods that minimize these adverse effects and preserve the integrity and performance of the vaccines to the greatest extent possible.^[49]^ In addition, due to the thin coating on the surface of the MNs, they have a small drug load and often need to concentrate drugs or vaccines before being coated (Figure 4d,l,m).^[34]^

Dissolving MNs are prepared from biodegradable materials that dissolve and release loaded biologically active substances, such as vaccines, after entering the skin. Compared to other types of MNs, dissolving MNs allow for long‐lasting drug delivery by controlling the dissolution time (Figure 4e,n,o).^[35]^ The dissolution time of MNs is usually related to the preparation materials and MNs structure design.^[50]^ Moreover, in addition to the geometric structure, MNs density and other parameters, the polymers used for dissolving MNs synthesis also affect the application effect because they may enhance the immunogenicity of the antigen.^[51]^ Currently, dissolving MNs have been used for the delivery of a variety of vaccines.

## 4 VACCINES DELIVERED BY MICRONEEDLES SYSTEM

Percutaneous immunization via MN systems has garnered significant attention in vaccine delivery research due to its unique advantages including minimal invasiveness, reduced pain, and robust immunogenic responses. Extensive studies have demonstrated that MN‐based vaccine delivery not only improves immunization efficacy but also enhances vaccine stability, positioning this approach as a highly promising platform for next‐generation vaccine applications.^[52]^ For MN vaccines, in addition to the structure affecting their function, the composition of the vaccines is also closely related to the effectiveness of the vaccines.

### 4.1 Influences of antigens on vaccines efficacy

Antigens are an important component of MN vaccines, which can induce a specific immune response in the body and form an immune memory to prevent disease. There are many types of antigens used in vaccines, such as glycoproteins, peptides, whole cells, and so on. Their quantity, purity, and stability will affect the effectiveness of vaccines.^[53]^ Therefore, the choice of antigens plays a decisive role in MN vaccines.

The antigens should have high immunogenicity. Studies have shown that when their antigenicity spectrum is similar to that of natural viruses, it can effectively induce a specific immune response and improve the immunogenicity.^[54]^ For MN vaccines against viruses that mutate easily, the ability to create cross‐protection is a measure of the vaccine's efficiency. It has been shown that the effect of cross‐protection (not only against target pathogens but also against other similar pathogens) is mainly related to the antigenic distance in the vaccine and to a lesser extent to the route of administration, and that both MNs administration and intramuscular injection can successfully induce cross‐protection.^[24]^ Recombinant antigen also has great application potential in this field. With adjuvants, recombinant antigen MN vaccines can induce high antigen‐specific IgG titers.^[55]^ For MN vaccines to treat cancer, preventing immune escape (evading recognition and attack by the immune system through a variety of mechanisms) from tumors is key. The high mutation rate of tumor cells may render single antigens ineffective, whereas whole tumor cell antigens can trigger multiple antigenic epitopes, eliciting more potent anti‐tumor immunity and long‐term immune memory.^[56]^ In summary, antigens determine the specificity and protective capacity of MN vaccines.

### 4.2 Modulating effects of adjuvants

Adjuvants can modulate the immune response in the body, and the addition of appropriate adjuvants to MN vaccines can increase the immunogenicity of antigens, improve vaccine efficacy^[57]^ and expand the scope of the protection of people.^[58]^ However, unlike the common intramuscular vaccines, the MN vaccines have more limitations in terms of the adjuvants that can be used. Alum adjuvant, the most widely used adjuvant in current vaccines, exhibits localized toxicity and can induce granuloma formation when administered intradermally. Consequently, its application in MN vaccines is not feasible.^[59]^ In addition, some advanced adjuvant systems are less stable and therefore may fail during MNs preparation.^[60]^ Moreover, due to the strong immune ability of the skin, there may be more adverse reactions such as excessive immunity caused by the adjuvant. Therefore, it is necessary to choose safer and stable adjuvants for MN vaccines.

With the development of immunology, more and more studies have been conducted on adjuvants for MN vaccines. Studies have shown that many water‐soluble or prepared as aqueous formulation adjuvants are safer because they can be cleared quickly from the site of delivery.^[61]^ For example, the half‐life in vivo of the fully water‐soluble unmodified CPG ODN adjuvant is less than 60 min,^[62]^ whereas the half‐life for intramuscular clearance of the water‐dispersed oil‐in‐water emulsion adjuvant, MF59, would be 42h.^[63]^ In addition, as researchers continue to elucidate signaling pathways, the development of adjuvants that transmit signals through pattern recognition receptors (PRRs) is increasing.^[64]^ Studies have shown that such adjuvants can effectively activate specific adaptive immunity.^[65]^ In some states, dual adjuvant combinations can significantly enhance the immune effect of vaccines by synergistically activating different immune signaling pathways, optimizing antigen presentation, balancing the Th1/Th2 response, and enhancing the local inflammatory response.^[66]^ In general, although adjuvants play an auxiliary and regulatory role, they also have a great impact on the immune effect of MN vaccines.

## 5 THE APPLICATIONS OF MICRONEEDLES IN VACCINES AND IMMUNIZATION

MNs were first conceptualized in 1976 as a promising strategy for transdermal drug delivery. However, their widespread application remained limited until the 1990s, primarily due to the absence of advanced microfabrication technologies necessary for their precise and scalable production.^[35]^ Since then, the advantages of transdermal immunization have been gradually discovered, and the application of MNs in the field of vaccines and immunization has attracted extensive attention from researchers.

### 5.1 Prophylactic vaccines

#### 5.1.1 Influenza

Influenza is a highly transmissible respiratory disease caused by viruses that can be seasonally prevalent in all age groups.^[67]^ Influenza viruses are negative‐stranded RNA viruses, and they can be classified according to their antigenicity into three categories: A, B, and C.^[68]^ Influenza A and B are the main types transmitted in the population. They express two main surface antigens: hemagglutinin (HA) and neuraminidase (NA).^[69]^ Genes expressing HA and NA are prone to point mutations that prevent the body's immune system from recognizing and protecting against them, allowing the new viruses to spread rapidly.^[70]^ Transdermal delivery of influenza vaccines via MNs is expected to achieve cross‐protection against influenza viruses and achieve better prevention than current vaccines.

MNs can mimic the natural infection of influenza by optimizing the preparation materials to allow sustained release of the vaccines for 1–2 weeks. These MN vaccines have been shown to increase the breadth and titer of antibodies, providing greater protection against a deadly flu challenge.^[71]^ Moreover, the intradermal delivery route of MNs increases the immunogenicity of the vaccine, and low doses of influenza vaccine can induce high levels of antibody response, allowing for dose savings while maintaining vaccine efficacy.^[7]^ A phase I clinical trial showed that delivery of influenza vaccine using dissolving MNs can induce the body to produce a similar percentage of seroconversion as intramuscular injection (all *p* > 0.01), which was significantly higher than that of the placebo (all *p* < 0.0001), and there were no treatment‐related serious adverse events (NCT02438423). In addition, transdermal immunization with MNs can be used with adjuvants to induce strong immune responses, amplifying the protective efficacy of the vaccines and providing cross‐protection against seasonal influenza.^[72]^ Finally, influenza vaccines delivered via the MNs route can provide long‐lasting protection against influenza viruses by generating a long‐term serological memory.^[73]^

#### 5.1.2 COVID‐19

Coronavirus disease 2019 (COVID‐19) is caused by Severe Acute Respiratory Syndrome Coronavirus 2 (SARS‐CoV‐2), which is highly infectious, pathogenic, and mutable.^[74]^ SARS‐CoV‐2 is a novel positive‐sense single‐stranded RNA coronavirus that consists of four major structural proteins, including the sarcomeric protein (S), nucleocapsid (N), membrane protein (M), and envelope protein (E).^[75]^ Among them, S proteins play a key role in SARS‐CoV‐2 infection and pathogenesis and are highly immunogenic; therefore, S proteins and their receptor‐binding domains (RBDs) are two major antigenic targets for the development of SARS‐CoV‐2 vaccines.^[76]^ However, current vaccine delivery methods have limitations such as poor compliance, and the MN systems are expected to be a better delivery method for SARS‐CoV‐2 vaccines.^[77]^

**FIGURE 5:**
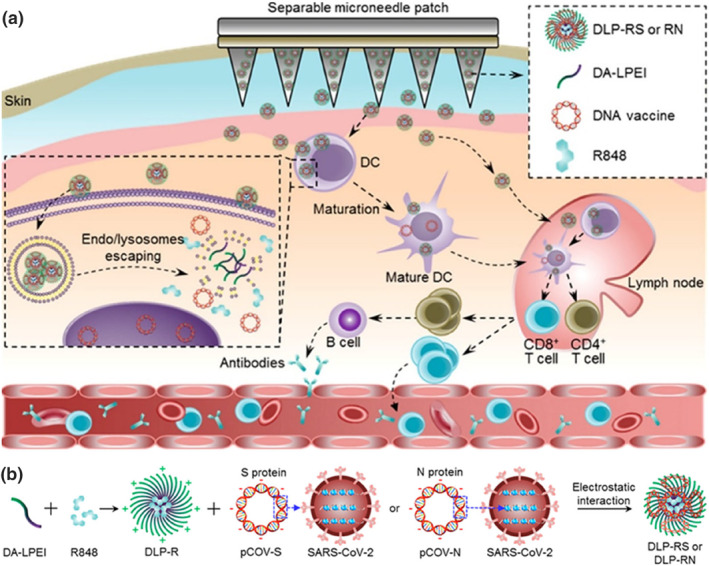
(a) Schematic illustration of separable MN patch mediated intracutaneous delivery of DNA nano‐vaccines for fighting SARS‐CoV‐2 virus. (b) DA‐LPEI amphiphilic polymer was designed to encapsulate the hydrophobic R848 in the core of nanoparticles (DLP‐R) and plasmid DNA for expressing S‐ or N‐protein (pCOV‐S or pCOV‐N) was absorbed on the nanoparticles through electrostatic interaction (DLP‐RS or DLP‐RN).^[79]^ Copyright 2021, American Chemical Society.

Unlike regular syringes, MNs can deliver vaccines while giving the skin other stimuli to boost immunity. The use of MN electroporation systems to deliver SARS‐CoV‐2 vaccines has been shown to induce high levels of antibodies, resulting in at least a tenfold reduction in dose compared with conventional intramuscular vaccines.^[78]^ Moreover, loaded SARS‐CoV‐2 vaccine MNs had higher stability. To address the challenges in the storage and transport of SARS‐CoV‐2 nucleic acid vaccines, Yin et al. reported an MN nanovaccine delivery system. The system adsorbed DNA vaccines encoding S or N proteins onto the surface of nanoparticles containing adjuvants (e.g., Resiquimod, R848) and administered intradermally via a separable microneedle (SMN) patch. The nanoparticles not only promote antigen presentation in DCs, but also help the DNA vaccine escape from endosomes/lysosomes and induce virus‐specific CD4/CD8 T‐cell responses and B‐cell antibody production. Furthermore, the MN nanovaccine can be stably stored at room temperature for at least 30 days without reducing the immune effect (Figure 5).^[79]^ In addition, like influenza viruses, SARS‐CoV‐2 viruses are also prone to immune escape by generating new variants. It has been shown that delivery of the SARS‐CoV‐2 subunit vaccines in high‐density MN patches can induce the production of antibodies that can neutralize the viral variants, thereby mitigating the impact of the viral variants on society.^[80]^

#### 5.1.3 Acquired Immunodeficiency Syndrome

Acquired Immunodeficiency Syndrome (AIDS) is caused by the Human Immunodeficiency Virus (HIV) of the genus Lentivirus and is usually transmitted through blood, mother‐to‐child and sex.^[81]^ HIV is roughly spherical in shape and can infect a variety of immune cells such as CD4 T cells, macrophages and microglia, causing immunodeficiency.^[82]^ HIV has spread worldwide but there is no effective HIV vaccine available for clinical use due to problems such as poor targeting and selectivity.^[83]^ However, MN‐mediated transdermal delivery of HIV vaccines has a higher safety profile and is expected to provide a new option for HIV vaccines.

Due to the short length of the MNs, they can be inoculated in the nasal cavity or mucous membranes, and this type of inoculation can induce large amounts of HIV vaccine antigen‐specific IgA, which can be very effective in preventing HIV transmission through the mucous membranes.^[84]^ In addition to mucosal immunity, MNs can successfully stimulate salivary IgA to neutralize HIV when administered in the mouth, reducing mother‐to‐child transmission of HIV during breastfeeding.^[85]^ Moreover, the MNs, when combined with nanoparticles, can enhance antigen delivery to cutaneous DCs, providing sufficient signals to trigger the T‐cell HIV‐1‐specific IFN‐γ response to HIV‐1, which is an efficient delivery route for HIV vaccines.^[86]^

### 5.2 Therapeutic vaccines (tumor therapy)

In recent years, active immunotherapy for tumors was being developed and investigated.^[87]^ Tumor vaccines eliminate cancer cells by activating the immune system to recognize and kill tumor cells, and they are mostly used for tumor treatment rather than prevention.^[88]^ Theoretically, therapeutic tumor vaccines only stimulate the body to produce specific immunity against the tumor without attacking normal cells, and they can induce a durable systemic immune memory to prevent tumor recurrence and metastasis.^[89]^ However, there are still some difficulties in using tumor vaccines in the clinic due to problems such as the high variability of tumor antigens and poor induced immune responses.^[90]^ As mentioned several times above, the MNs transdermal immunization route has an immune‐enhancing effect and could similarly be used for tumor vaccines, making it a potential delivery route for tumor vaccines.

**FIGURE 6:**
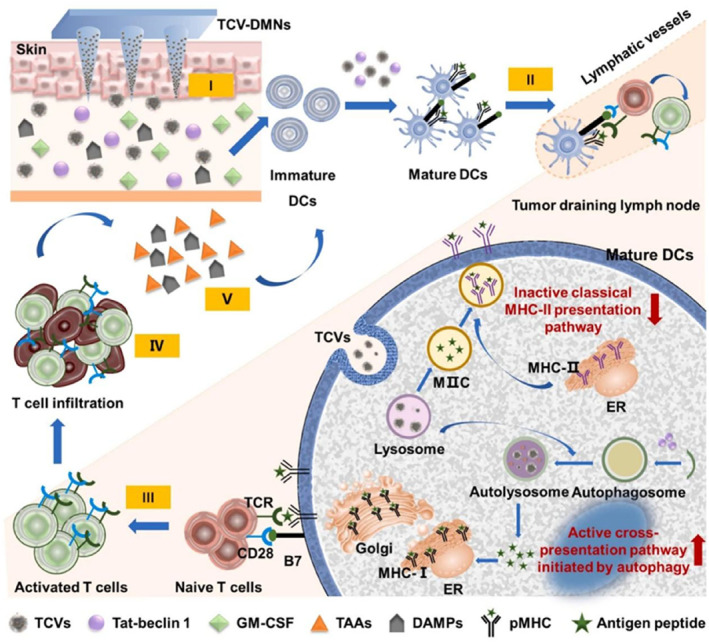
TCV‐DMNs for antitumor immunotherapy. Cancer‐Immunity Cycle: I. Tumor antigen release; II. Tumor antigen is presented by DCs; III. Activate effector T cells; IV. T cell infiltration in tumor tissues; V. T cells kill tumor cells and release tumor antigen.^[91]^ Copyright 2023, Elsevier B.V.

MN‐mediated transdermal delivery can target vaccines to DCs, which can improve tumor treatment efficacy and prolong survival time compared with non‐DC targeting methods (Figure 6).[^91^, ^92^] The strong immunity delivered transdermally in combination with immunotherapeutic agents is effective in increasing treatment efficacy and reducing side effects. The MNs prepared by Zheng et al. enable slow intradermal release of OVA, which can trigger a stronger and longer‐lasting adaptive immune response than conventional injections.^[93]^ Chang et al. used MNs loaded with both living DCs vaccine and immune checkpoint inhibitor (PD‐1) monoclonal antibody to induce a higher antigen‐specific cellular immune response.^[94]^ Moreover, transdermal delivery of immunostimulants with MNs is also effective in reducing tumor recurrence.^[95]^ In addition to immune modulation, the same enhancement of vaccine stability by MNs also applies to tumor vaccines. The instability of mRNA limits its application in the field of tumor vaccines, but when they are prepared into MNs, they can be stored for at least 2 weeks without degradation and induce stronger immune responses than subcutaneous injection.^[96]^ In addition, MNs enable smart vaccine delivery, which can achieve controlled release of vaccines through responsive copolymers, maintain the durability of vaccine effects, effectively activate cellular and humoral immunity, and inhibit tumor growth and metastasis.^[97]^ In addition to delivering the vaccines themselves, the MNs can deliver photothermal nanoparticles to the tumor site to form in situ vaccines, which can induce immunogenic cell death (ICD) (release of signals upon cell death that activate the immune system to launch an attack against pathogens or abnormal cells) through the synergistic amplification of the photothermal effect, to achieve the safe treatment of primary tumors.^[98]^ The use of MNs in oncology is still in its early stages, but its potential for therapeutic vaccines and immunotherapy is enormous.

### 5.3 Other immunological treatments

Due to the small size and high stability of MNs, they can be used for other immunological treatments in addition to vaccine delivery. Firstly, as MNs are a topical treatment on the skin, they can be used to treat allergies through Allergen‐Specific Immunotherapy (AIT) and prevent causing hypersensitivity reactions. MN‐assisted epidermal immunotherapy (EPIT) treats allergies by depositing allergens into the skin, stimulating immune cells, and achieving sustained allergen exposure to the skin, resulting in allergen tolerance.^[99]^ A clinical trial using MN patches to screen for biomarkers of immunotherapeutic response in patients with Allergic Rhinitis (MIST) is enrolling subjects to evaluate the prognosis of immunotherapy for this allergic disease (NCT05922176). Secondly, MNs can not only be delivery systems but also biosensors. They can specifically capture antibodies in the skin interstitial fluid by antigen‐antibody binding after insertion into the skin for in vitro examination, which can be used for efficient detection of antibodies after vaccination.^[100]^ Finally, MN‐mediated transdermal delivery could not only enhance immunity but also regulate the excessive immune response in the skin by loading anti‐inflammatory substances to treat skin‐related immune diseases.^[101]^

## 6 CONCLUSION AND FUTURE PROSPECT

MNs represent a versatile transdermal delivery platform capable of being fabricated from diverse materials through various manufacturing techniques. The structural and compositional variability among MNs enables distinct delivery mechanisms, offering flexibility for tailored applications. Given the abundance of tissue‐resident immune cells, such as LCs, in the skin, MNs have garnered significant attention in vaccine delivery and immunotherapy. Their use not only enhances vaccine stability and immunogenicity but also reduces required dosages and broadens immunization coverage across diverse populations. These attributes position MNs as a promising tool for the delivery of prophylactic vaccines, tumor immunotherapies, and treatments for a range of immune‐mediated diseases, underscoring their substantial advantages in modern biomedicine.

However, they still need to be improved and optimized in a number of areas before they can be applied on a large scale and fully commercialized. Firstly, because of the immune properties of skin tissue, MN vaccines may cause unwanted immune response results such as over‐immunization. Therefore, how to regulate skin immunity while improving vaccine efficacy through adjuvants is a problem that needs to be considered. Secondly, although MN patches can enhance the stability of vaccines, the processing may lead to the loss of vaccines efficacy, so perfecting the preparation process is essential in the future development of MN vaccines. Thirdly, exploring the appropriate dose of percutaneous immunization in different populations is also a necessary study. Finally, with the development of MNs as an emerging delivery method, the state regulatory system for this medical device needs to be refined. As research efforts to address these challenges advance, MN vaccines are poised to emerge as a pivotal platform for vaccination in the future.

## CONFLICT OF INTEREST STATEMENT

The authors declare no conflicts of interest.
